# Impact of implant abutment materials on force damping response and marginal fit of implant supported restoration

**DOI:** 10.1186/s12903-025-06112-0

**Published:** 2025-05-20

**Authors:** Amr Mohammed Hussein Elmesery, Amany Mohammed Korsel, Waleed Elshahawy

**Affiliations:** 1 Fixed Prosthodontics Department, Faculty of Dentistry, King Salman International University, South Sinai, Egypt, Egypt; 2 Fixed Prosthodontics Department, Faculty of Dentistry, Tanta University, Tanta, Egypt

**Keywords:** Implant Abutment, Force Damping Response, Marginal Fit, Cementation, Dental Restoration

## Abstract

**Background:**

The marginal fit and force-damping response of implant-supported restorations play critical roles in the long-term success of dental implants. This study evaluates the effect of implant abutment materials— resin-ceramic material, lithium disilicate, PEEK, and Titanium- on implant-supported restorations' marginal fit and force-damping response. The study offers novel insights into stress distribution and marginal gaps, aiming to optimize implant-supported restoration outcomes.

**Methods:**

Forty implant abutments were divided into four equal groups: Shofu HC, Tessera, BioHPP, and Titanium. Vertical marginal gap measurements were taken using a digital microscope before and after Cementation, and force damping was assessed using a custom impact test machine. Non-metal abutments were custom-fabricated using STL files and a CAD/CAM machine (CEREC MC X5, Dentsply Sirona) for Tessera (MT/LT-BL2), Shofu HC Block (A3-LT/M), a resin hybrid ceramic (61% zirconium silicate, 39% nano-filler composite), and BioHPP (bredent GmbH & Co KG). Quantitative data were expressed as mean ± SD and analyzed using ANOVA with post hoc Tukey test. Normality was confirmed with the Shapiro–Wilk test, and differences between groups were assessed with an unpaired Student's t-test.

**Results:**

Before Cementation, the Biohpp group demonstrated the highest marginal gap (35.49 ± 2.31 µm), followed by Titanium (31.05 ± 1.87 µm) and Shofu HC Block (29.35 ± 1.72 µm). Tessera exhibited the lowest marginal gap (23.70 ± 2.99 µm) (*P* < 0.001). After Cementation, marginal gaps increased across all groups, with Biohpp (46.47 ± 3.10 µm) and Titanium (38.43 ± 2.25 µm) showing the most significant gaps, while Tessera continued to demonstrate the lowest (30.80 ± 1.64 µm) (*P* < 0.001). In force damping tests, Shofu HC Block recorded the lowest impact force (0.804 ± 0.034 N), followed by Biohpp (0.866 ± 0.027 N) and Tessera (0.920 ± 0.029 N). Titanium exhibited the highest force (0.970 ± 0.033 N), with all results showing statistical significance (*P* < 0.001).

**Conclusions:**

Lithium disilicate exhibited the smallest marginal gap before and after Cementation, while PEEK showed the largest, followed by Titanium and resin-ceramic material. Resin-ceramic material had the highest shock absorption for force damping, followed by PEEK and Lithium disilicate, while Titanium recorded the highest impact force, indicating the least damping ability.

## Background

Implant-supported prostheses have emerged as a highly effective therapeutic option for wholly or partially edentulous patients [1]. However, these prostheses are more prone to occlusal overloading than tooth-supported crowns due to the absence of a periodontal ligament between the tooth and bone. As a result, managing stress distribution becomes crucial in ensuring the longevity and functionality of these restorations [2].

Although many studies have examined stress distribution in dental implants, this research compares the performance of different abutment materials—resin nanoceramic, lithium disilicate, poly-ether-ether-ketone (PEEK), and the more traditional Titanium. The selection of abutment material is vital for managing occlusal loads and optimizing stress distribution across the implant-abutment-bone complex, significantly impacting implant-supported restorations'success [3, 4].

Resin ceramic materials, such as Shofu HC Block, offer numerous advantages in implant-supported restorations due to their unique composition, which combines polymer matrices with ceramic components. These materials improve shock absorption and force distribution, which helps to reduce the stress transferred to the peri-implant bone. Their lower modulus of elasticity compared to traditional ceramics enhances their resilience, contributing to the long-term success of implant-supported restorations [5]. Their machinability and superior marginal fit further increase their viability for improving the biomechanical performance of prosthetic restorations [6, 7].

The recently developed CAD-CAM material, CEREC Tessera, is a lithium disilicate-based material composed of 0.5 μm in length crystals. These crystals are embedded in a glassy matrix and lithium aluminum silicate crystals, platelet-like and measure 0.2–0.3 μm, with a biaxial flexural strength of approximately 700 MPa [8]. This material has demonstrated excellent marginal fit before and after heat treatment, making it suitable for clinical use [9–11].

PEEK, a thermoplastic linear homopolymer, is another promising abutment material known for its exceptional performance. Due to its unique properties, PEEK reduces stress on the implants and minimizes stress shielding [3]. BioHPP PEEK, a variation of PEEK, can be fabricated using CAD/CAM technology through milling or pressing granular PEEK material. This material significantly reduces the magnitude of masticatory forces in vertical and lateral directions compared to Titanium, zirconium, or ceramic materials. Its low modulus of elasticity, similar to that of human bone, allows it to distribute stress more evenly to the surrounding tissues, helping to preserve the health of peri-implant bone [12, 13].

Titanium, known for its excellent biocompatibility and mechanical properties, remains one of the most commonly used materials for implant abutments. However, titanium abutments are limited, particularly in aesthetics and force distribution [14].

Ceramic abutments are typically connected to the implant body using titanium bases, resulting in a direct interface between titanium materials instead of a direct connection between ceramic and Titanium components. This can influence the restoration's force distribution and overall biomechanical behavior [15].

Moreover, implant loading directly transfers stresses onto the peri-implant bone, as there is no shock-absorbing component to buffer these forces. Overloading can lead to bone modeling changes or even resorption and fractures if pathological overload occurs. Therefore, the shock absorption capabilities of implant-supported restorations are essential in compensating for the loss of the periodontal ligament, improving the restoration's overall success [16].

Moreover, implant loading directly transfers stresses onto the peri-implant bone, as there is no shock-absorbing component to buffer these forces. Overloading can lead to bone modeling changes or even resorption and fractures if pathological overload occurs. Therefore, the shock absorption capabilities of implant-supported restorations are essential in compensating for the loss of the periodontal ligament, improving the restoration's overall success [17].

By evaluating both the force-damping response and marginal gaps, this study provides valuable insights into how different abutment materials influence the biomechanical behavior and overall performance of implant-supported restorations. These findings could guide future research and clinical decisions regarding the optimal selection of abutment materials for implant-supported prostheses.

This study aimed to assess the impact of implant abutment materials on force damping response and implant-supported restorations marginal fit.

The null hypothesis tested was that implant-supported restorations'force-damping response and marginal fit wouldn't show significant differences among different abutment materials.

## Methods

### Sample size calculation

The required sample size for this study after calculating the dropout rate is 40 samples. The sample is collected based on a previous study [18]. The significance level was 0.05, the power sample size was more than 80% for this study, the confidence interval was 95%, and the actual power was 96.58%. The sample size and the effect size = 0.82915, and the sample size is calculated using a computer program called G*Power 3.1.9.2 (Kiel University).

The materials used in this study are listed in Table 1.

**Table 1:** The materials that were used in the study

Material	Trade name	Composition	Manufacture
Polymeric Biomaterial	Biohpp (Biocompatible High-Performance Polymer), LOT No. 484123	- 70–80%Polyetheretherketone - 10–20%Hydroxyaptite - 5–15%5Barium sulphate 0–5%Other trace elements	- Bredent Gmph&KG
Hybrid (resin nano ceramic)	Shofu HC, LOT No. 0217385	- 61% zirconium silicate 39% Nano filler composite	- SHOFU INC
Advanced lithium Disilicate	CEREC Tessera, LOT No. 16008745	- 50–60% feldspar - 20–30% glass - 10–20% quartz - 5–10%Lithium disilicate 0–5% other trace elements	- Dentsply Sirona
Titanium Dental Implant	OXY Implant, LOT No. 2180569	98%Titanium alloy base 2% titanium oxide	OXY dental implants
Dual cured Self Adhesive Resin Cement	Nova Resin Cement, lot No C378	BisGMA, urethanedimethacrylate, and triethylene glycol dimethacrylate. The inorganic fillers are barium glass, ytterbium trifluoride, Ba-Alfluorosilicate glass, and spheroid mixed oxide Additional contents: initiators, stabilizers and pigments	IMICRYL
Polycrystalline Zirconia ceramic	Katana Ultra Translucent Multi-layered (UTML)	Hafanium oxide and zirconium oxide (ZrO2 + HfO2):. 87–92% Yttrium oxide (Y2O3): 8–11% Other oxides: 0–2%	Kuraray Noritake Dental Inc

This experimental laboratory work was performed on 40 specimens; we divided the specimens into four groups equally depending on implant abutment material: resin-ceramic material: Shofu HC group, lithium disilicate: Tessera group, PEEK: Biohpp group, and Titanium (control group).

### Specimens’ preparation

Abutment fabrication: Scanning of the titanium stock abutment with 5.5 mm width, 4 mm gingival height, and 6 mm length. D scan spray (Dentify GmbH, Germany) achieves optimal accuracy and results with 3D laser scanning. Neway 3D scanner ScanWay software (biomil dental products, Australia) is used for abutment scanning.

**Fig. 1:**
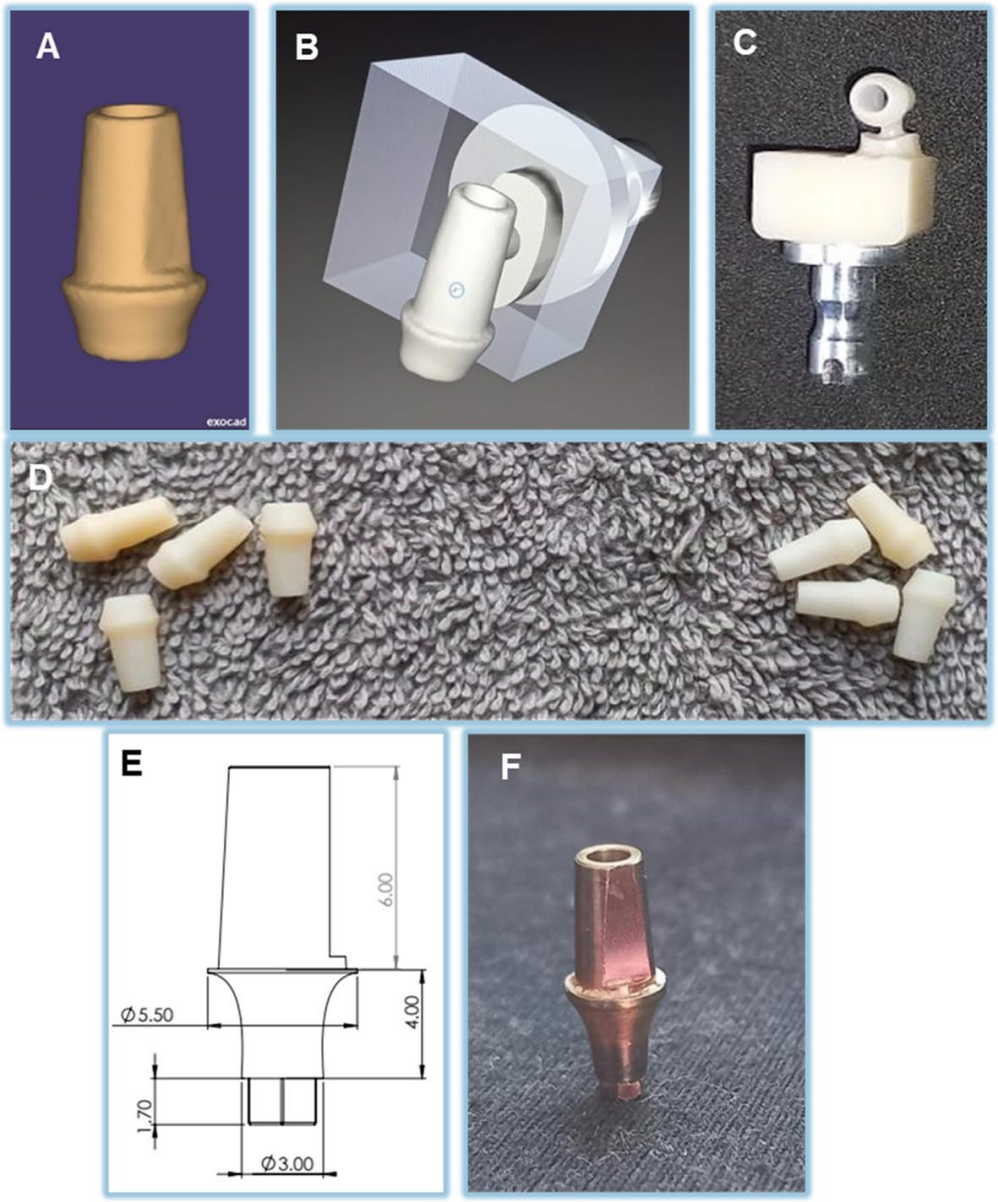
Custom made non-metal abutments fabrication & stock titanium. (**A**) View of STL file (**B**) View of custom non-metal abutment before milling (**C**) View of Custom non-metal abutment after milling (**D**) Ready-to-use Custom non-metal abutments of different materials (E) Titanium stock abutment dimensions (**F**) Titanium abutment before scanning

The Standard Tessellation Language file (SLT) format is a publicly published format used to describe an object surface using a triangular mesh. STL file was made for the stock abutment to make custom non-metal abutments with the exact dimensions of the stock titanium one (Fig. 1).

Custom-made non-metal abutments were fabricated using STL files and a CAD/CAM machine (CEREC MC X5, Dentsply Sirona) for Tessera (MT/LT-BL2)(Dentsply Sirona), Shofu Block HC (A3-LT/M 10 mm × 12 mm × 16 mm) (Shofu Dental Corporation), a resin hybrid ceramic composed of 61% zirconium silicate and 39% nano-filler composite, and BioHPP (bredent GmbH & Co KG). The Ti-base abutments and implant system utilized for the study were provided by OXY Implants (Biomec SRL, Colico, Italy), ensuring precise implant-abutment connections.

### Cementation of custom abutments to titanium bases

**Fig. 2:**
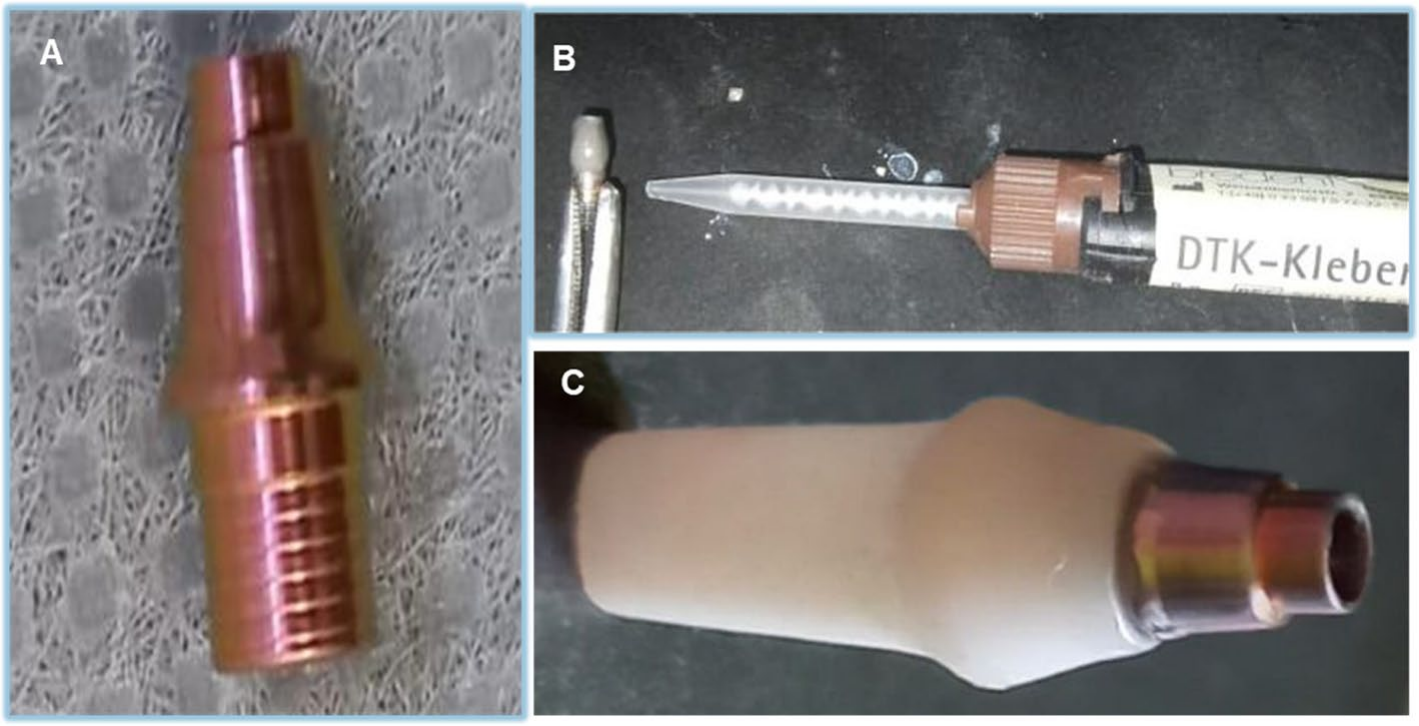
Cementation of abutment to titanium base. (**A**) Titanium base (**B**) resin cement application on Ti-base (**C**) Cemented abutment on Ti-base

The titanium bases were roughened by airborne particles utilizing 110 µm aluminum oxide particles at 2 bar pressure and 10 mm distance, and the Tessera (lithium disilicate) abutments were etched with 9.5% hydrofluoric acid (BISCO) for 15 s, rinsed, and treated with a silane coupling agent (BISCO) for 1 min. No surface treatment was applied to the Biohpp and Shofu HC abutments. The screw channels were sealed with wax, and (Visiolink, Bredent GmbH & Co. KG) bonding agent was applied to the titanium bases. DTK-adhesive (Bredent GmbH & Co. KG) was used for the titanium base and the custom abutment to ensure a strong bond. The abutments were pressed onto the adhesive base, and temporary screws were inserted into the channels before polymerization to maintain proper alignment. Polymerization was performed using the Bre.Lux Power Unit2 (Manufacturer: Bredent GmbH & Co. KG), 370–500 wavelength, 20 s duration. After polymerization, the screws were removed, and any excess cement was carefully cleared (Fig. 2).

### Crown fabrication

Zirconia crowns of mandibular first molars were fabricated using Neway 3D scanner ScanWay software used for abutment scanning and detecting the abutment margins; a cement gap of 0.05 mm from the margin was set using Neway 3D scanner software to ensure a precise fit. The occlusal surface of the crowns was designed to accommodate the steel ball with a 12 mm diameter used in the impact test machine. The buccolingual dimensions of the crowns were 8.5 mm, mesiodistal dimensions of the crown 10.7 mm, occluso gingival height 9 mm from the proximal aspect, occluso gingival height from the buccal view 9.7 mm, and Zirconia crowns were dry milled from 5.4 mol% yttria-stabilized zirconia discs (monolithic ultra-translucent multi-layered Zirconia (UTML), KATANA™ UTML. Zirconia, Kuraray Noritake Dental Inc) utilizing a 5-axis milling machine (Roland DWX 52D, DGA Corporation) and then sintered following the manufacturer’s instructions at 1520 °C ultimate temperature for 8 h (Sintering furnace: Tegra SPEED. Yenadent).

Throughout the dry process of milling, a suction device coupled to the milling (CAM) system was used to collect the milling chips. The frameworks were extracted from the disk using a diamond bur following the milling process. The residual cutting waste or dust adhered to the crowns was eliminated using a gentle stream of air steam. The restorations were inserted into the refractory tray and placed in the sintering furnace.

**Fig. 3:**
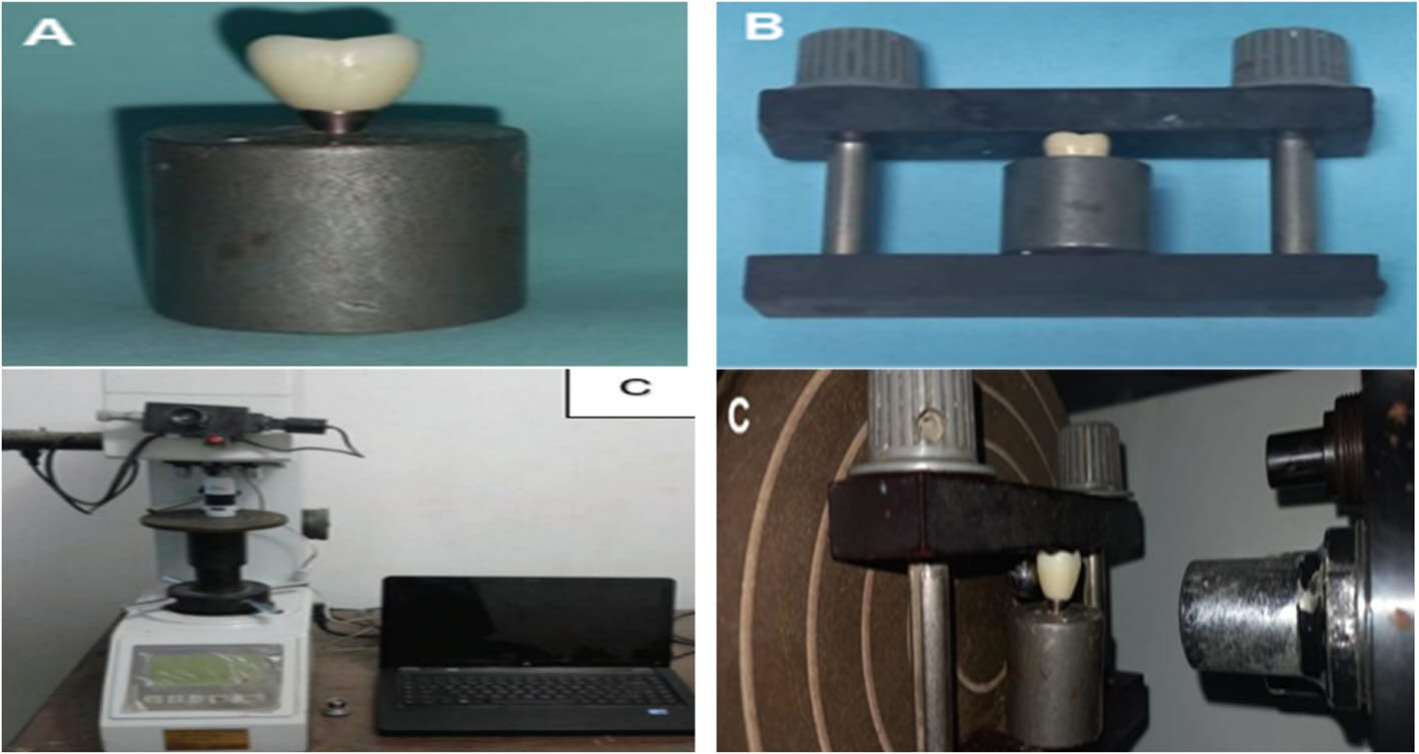
Evaluation of marginal adaptation. (**A**) Metal base (**B**) Specially designed holding device (**C**) Sample under digital microscope

Evaluation of marginal adaptation before Cementation: The specimens were secured above the metal base using a specifically designed holding device that securely held the specimen while taking shots of the margins. All aspects were predetermined using equidistant 3 points in each element, one in the middle and two correct and left to the middle point): The specimens were photographed using a USB Digital microscope with a built-in camera. The images were taken using the following image acquisition system: 1) Digital camera (U500x Digital Microscope, Guangdong, China) with 3 Mega Pixels of resolution, placed vertically at a distance of 2.5 cm from the samples. The angle between the lens axis and the illumination source is approximately 90°). Illumination was achieved with 8 LED lamps (Adjustable by Control Wheel), with a color index close to 95%. The images were taken at maximum resolution and connected to an IBM-compatible personal computer using a fixed magnification of 40X. The images were recorded with a resolution of 1280 × 1024 pixels per image (Fig. 3).

### Crown cementation

**Fig. 4:**
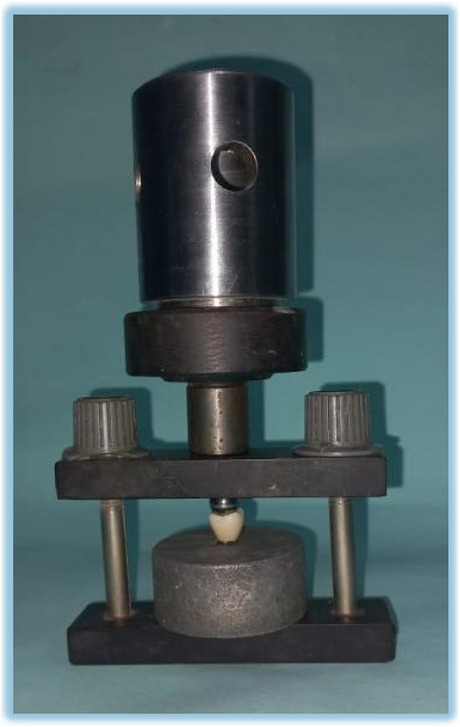
Custom made seating device

For Cementation standardization, we used a custom-made seating device with a load (3 kg) for 5 min to ensure crown seating for all specimens. The specimens were secured above the metal base using a specifically designed holding device that securely held the specimens. Crown fitting surfaces were surface treated by sandblasting (10 mm distance) with 110 µ Alminuim with 2 bar pressure and then cleaned with steam air. Nova self-adhesive resin cement (IMICRYL Fetih Mah. Mahir Sok) was applied to the fitting surface and inserted into a cavity of the corresponding crown. Foam pellets removed excess cement. Polymerization was achieved for 20 s on each surface (Fig. 4).

### Evaluation of marginal gap distance after Cementation

**Fig. 5:**
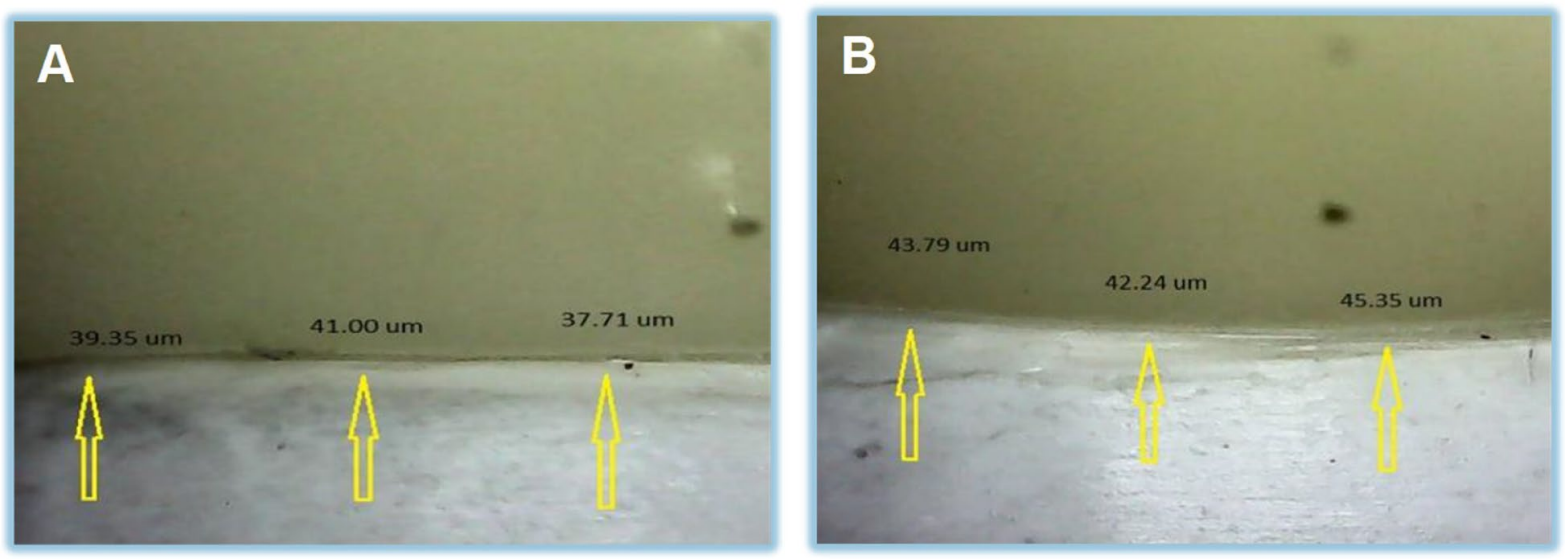
Evaluation of marginal gap distance. (**A**) Before Cementation (**B**) After Cementation

A digital image analysis system (Image J 1.43U, National Institute of Health, USA) was used to measure and evaluate the gap width. All limits, sizes, frames, and measured parameters are expressed in pixels within the Image J software. Therefore, system calibration converted the pixels into absolute real-world units. Calibration was made by comparing an object of known size (a ruler in this study) with a scale generated by the Image J software. Each crown was seated on the corresponding original crown preparation, stabilized externally, and then positioned in the device's base. On each surface, three equidistant markings (grid lines) at the three measurement locations were done using image-J software to standardize crown positioning and measurement points. Shots of the margins were taken for each specimen. Then, morphometric measurements were done for each shot. The microscope was linked to an IBM-compatible personal computer, and a fixed magnification of 50X was used. A consistent digital image analysis technique was utilized to quantify and assess the length of the gap (Fig. 5). The specimens were secured using a custom-made holding mechanism. Photographs of the margins were captured for each sample with predetermined equidistant three points, one in the middle and two correct and left to the middle point.

### Force damping response test

**Fig. 6:**
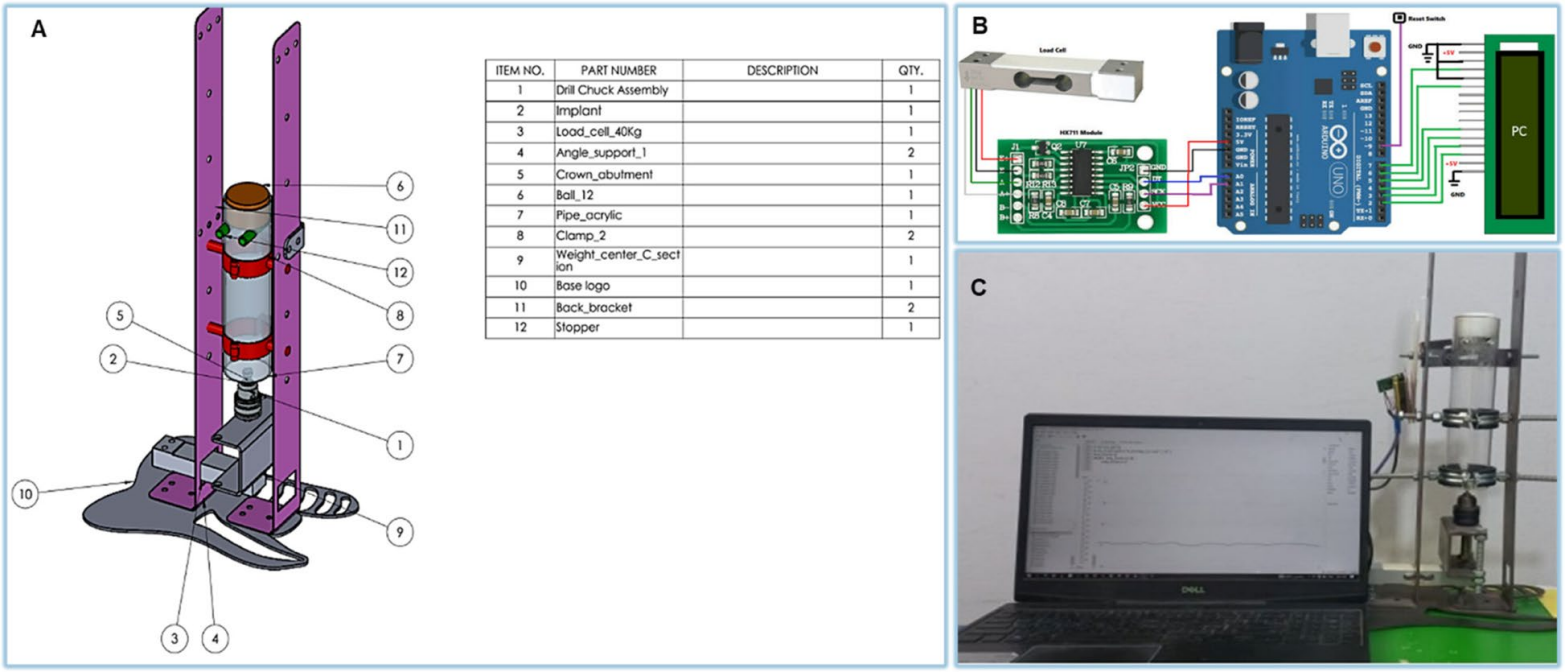
specially designed impact test machine (**A**) impact test machine design (**B**) The HX711 a precision 24-bit analogue to convert the analogue signal (**C**) impact test machine

A specially designed impact test machine was created using SOLIDWORKS software to measure the resulting impact force (Fig. 6). Because the universal testing machine's load is gradual, not an impact load, it is essential to do an impact test and detect the force-damping response. Load weight and height were detected after making a few trial and error tests to reach to weight and height which didn’t cause fracture and didn’t exceed the yield strength of the specimens to accurately detect the force damping response of the different abutment materials. The machine included an aluminum bar load cell weighing 40 kg and the HX711. This precision 24-bit analog-to-digital converter converts the signal from the load cell to digital form. The Raspberry Pi Pico controller was selected for its high speed and compatibility with PCs for viewing and recording data. **PicoPython software** was used to calibrate the machine, ensuring accurate measurements. The calibration process involved multiple trial tests to adjust the load weight and height, ensuring the impact forces were within the material's yield strength. Specimens were placed centrally on the specially designed impact machine. The free-fall drop test was conducted using a 12 mm diameter stainless-steel ball attached to an additional load, giving a total weight of (61.5 g), dropped from a height of (18 cm). The machine’s calibration was confirmed by comparing the results with standard reference measurements, ensuring accuracy [19, 20].

### Statistical analysis

Statistical analysis was conducted using SPSS v28 (IBM Inc., Armonk, NY, USA). Quantitative data were presented as mean and standard deviation (SD) and compared across groups using the ANOVA (F) test, followed by a post hoc Tukey test. The Shapiro–Wilk test was used to assess the normality of the data, which confirmed a normal distribution. The unpaired student t-test was used to compare the two groups in quantitative data. A satisfactory power level of 80% was set, with a 95% confidence interval. A two-tailed *P*-value of less than 0.05 was considered statistically significant.

## Results

**Fig. 7:**
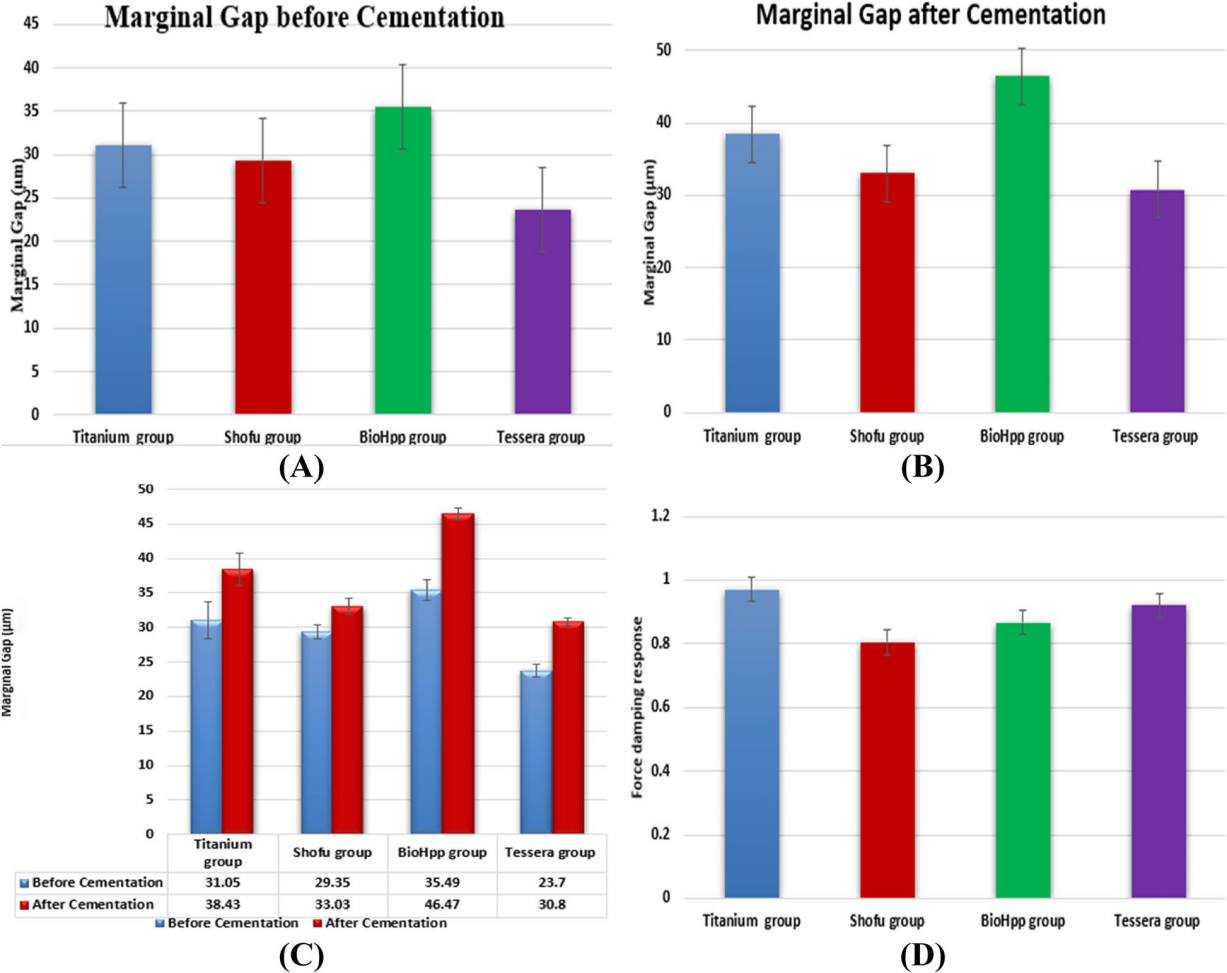
Marginal Gap (**A**) Before and (**B**)after Cementation, (**C**) Comparison between Marginal gap before and after Cementation and (**D** Resulting force) of the studied groups

Table 2 and Fig. 7 show the marginal gap results Before and after Cementation for the studied groups, expressed by mean, standard deviation, and range. It also shows the comparison between before and after Cementation in each group using paired t-test, which showed there is no significant difference between before and after in the titanium group with a *p*-value of 0.066, where there is a significant difference between before and after at Shofu HC group with *p*-value 0.021*, since there is a highly significant difference between before and after Cementation at BioHpp group with *p*-value 0.000**, also at Tessera group there is a highly significant difference between before and after Cementation with *p*-value 0.000**. The comparison between the groups using a one-way ANOVS test showed a highly significant difference with a *p*-value of 0.000** for the force-damping response test.

**Table 2:** Marginal gap before and after Cementation and resulting force between studied groups

		Marginal Gap before Cementation (µm)	Marginal Gap after Cementation (µm)	Comparison between before & after	Resulting force (N)
				*P*-value	
Shofu HC group	$\overline{X }$±S. D	29.35 ± 3.28	33.03 ± 3.65	0.021*	0.804 ± 0.034
	Min–Max	24.43—35.71	27.22—37.22		0.750—0.845
Tessera group	$\overline{X }$±S. D	23.70 ± 2.99	30.80 ± 1.64	< 0.001**	0.920 ± 0.041a
	Min–Max	20.56—30.29	27.86—32.93		0.858—0.974
BioHpp group	$\overline{X }$±S. D	35.49 ± 4.78ab	46.47 ± 2.73ab	< 0.001**	0.866 ± 0.035ab
	Min–Max	27.38—41.11	42.25—50.34		0.805—0.907
Titanium group	$\overline{X }$±S. D	31.05 ± 8.34b	38.43 ± 7.52abc	0.066	0.970 ± 0.045abc
	Min–Max	17.56—43.46	23.11—46.53		0.906—1.039
ANOVA test		< 0.001**	< 0.001**	––––-	0.001**

Data presented as mean $\overline{{\varvec{X}} }$±S. D

^a^*P* value compared to Shofu HC group

^b^*P* value compared to Tessera group

^c^*P* value compared to BioHpp group

^*^Significant *p* value < 0.05

^*^Significant *p* value < 0.05

^**^Highly significant *p* value < 0.001

Table 3 examines the difference between before and after Cementation in each group for a marginal gap. A paired t-test showed no significant difference between before and after in the titanium group, with a p-value of 0.066. In contrast, there is a significant difference between before and after in the Shofu HC group with a *p*-value of 0.021*, since there is a highly significant difference between before and after Cementation in the BioHpp group with a *p*-value of 0.000**, also at Tessera group there is a highly significant difference between before and after Cementation with *p*-value 0.000**. There is a highly significant difference between the studied groups with a *p*-value of 0.000**for the Force damping response test results.

**Table 3:** The multiple comparison Tukey test

	**Shofu HC vs. Tessera**	**Shofu HC vs. BioHpp**	**Shofu** **vs. Titanium**	**Tessera vs. BioHpp**	**Tessera** **vs. Titanium**	**BioHpp vs. Titanium**
Marginal Gap before Cementation	0.057	0.033*	0.860	< 0.001**	0.008*	0.184
Marginal Gap after Cementation	0.617	< 0.001**	0.025*	< 0.001**	0.001*	< 0.001**
Resulting force	< 0.001**	0.007*	< 0.001**	0.020*	0.038*	< 0.001**

^*^Significant *p* value < 00.05

^**^Significant *p* value < 0.001

## Discussion

This study aimed to assess the impact of implant abutment materials on the force-damping response and marginal fit of implant-supported restorations.

The hypothesis was rejected because there was a significant difference between marginal fit and force damping response of different implant abutment materials.

In agreement with our results about the marginal gap before Cementation, Hegazy et al. [21] found that the highest mean ± SD values of Marginal gap were recorded for the PEEK group (76.13 ± 2.88 μm) followed by Titanium group mean ± SD values (56.61 ± 2.79 μm). The group variation was statistically significant, as detected using one-way ANOVA followed by Tukey’s pair-wise post-hoc test (F = 276.9, *P* < 0.001). Also, our findings aligned with several previous studies by Sukkasam [22]. The findings of this work align with Martinez-Rus et al. [23], which showed that, before Cementation, they showed significantly greater mean marginal openings on both Titanium and PEEK groups (75.2 ± 12 and 77.5 ± 13 μm, respectively).

In agreement with our results about the marginal gap after Cementation, Martinez-Rus et al. [23] found that, after Cementation, both Titanium and Zirconia showed significantly larger marginal gap values than other groups.

The results showed that the resin-ceramic material group documented a statistically significant higher mean value of the marginal gap after Cementation.

The current study results observed that the Tessera group documented statistically significant more excellent mean value of the marginal gap after Cementation than before, as proved by paired t-test.

The present study findings showed that the Biohpp group recorded a statistically significant higher marginal gap mean value after Cementation than before, as proved by paired t-test.

According to our findings, the Titanium group recorded a statistically significant higher marginal gap mean value after Cementation (38.43 µm) than before (31.05 µm), as proved by paired t-test with a *p*-value = 0.0437. The findings of this work align with those of Hegazy et al. [21], who found that the resin-ceramic material group documented a greater mean ± SD value of marginal gap after Cementation than before Cementation mean ± SD value. An additional recent study by Zahoui et al. [24] noted that before and after Cementation, Titanium did not record any significant difference.

We can explain the difference in the marginal gap between each material's composition and manufacturing methods as follows: PEEK abutments exhibit a significant vertical displacement compared to titanium abutments. Additionally, they show plastic deformation at the abutment-implant interface. PEEK abutments could be suitable for implant restorations, particularly in the anterior region and for patients without parafunction. However, considerations such as torque loss and microleakage are important factors to address when using PEEK abutments [14].

Prefabricated resin ceramic materials made using nanoceramic resin technology offer combined advantages of both ceramic systems and composite resins [11]. These nanoceramic resin materials are known for their excellent marginal fit, making them a good option for implant restorations.

Lithium disilicate is an operator-friendly material that achieves high marginal adaptation.

It has demonstrated the best marginal fit both before and after heat treatment, making it an excellent choice for applications where precision is crucial [25].

In agreement with our force-damping results, Taha & Sabet [26] reported that implant-supported crowns'force-damping characteristics vary depending on the material used. However, crowns made from resilient materials like polymer-infiltrated ceramics and PEEK demonstrate superior force absorption compared to stiff materials, which include zirconia and lithium disilicate ceramics. Previously, Menini et al. [17] noted that Zirconia and ceramic crowns exhibited higher force peaks than other materials. These impacts were attributed to the varying elastic moduli of the studied materials. In a previous study, Della-Bona et al. [27] reported that the data obtained supported the rejection of the null hypothesis, as there were statistically significant differences in the force-damping behavior of implant-supported crowns for different crown materials and luting agents. The modulus of elasticity describes a material's relative stiffness or rigidity and, therefore, is crucial in determining how a material behaves when subjected to stress. In a previous study, Rosentritt et al. [18] reported that there were significant variations (*P* < 0.001) in the applied and resultant forces among the crown materials that were not cemented, temporally cemented, cemented, and bonded adhesively.

We could explain the difference in force damping response that titanium dental implant abutment materials have traditionally lacked shock absorption capabilities, which can lead to increased stress on the bone-implant interface. PEEK is a semicrystalline linear polycyclic aromatic polymer that has been claimed to be a suitable material for dental applications with stress distribution to surrounding tissues because of its low modulus of elasticity, which is close to human bone. [3] Because of their higher polymeric content and lower elastic modulus of resin ceramic materials compared to ceramics, resin ceramic materials exhibit improved biomechanical behavior for implant-supported reconstructions. This results in favorable resilience and improved occlusal forces damping and shock absorption (68, 85). On the other hand, Lithium disilicate materials have high fracture resistance. They can, therefore, withstand higher loads and transfer stresses to the components of the implant complex because of their high stiffness. [28, 29]

The limitations of this study included the fact that different abutment materials are essential. One crown type was used in the study as cement selection can play a significant role in the overall performance of the implant-supported restoration. Different types of cement (e.g., resin-based, glass ionomer, or zinc oxide-eugenol) have varied bonding strength, elasticity, and thickness properties, which can affect marginal fit and stress distribution. Additionally, one cement type was used in the study. No Aging was made before marginal gap evaluation or force damping response test that may not reflect the actual performance of the materials in a real-world, long-term clinical setting, where materials may degrade or change over time. While surface treatments were applied to lithium disilicate, they were not performed on PEEK or resin-ceramic materials. These untreated materials may have had different bonding characteristics or stress responses than those that underwent treatment—the study's reliance on in vitro conditions may not fully replicate clinical settings. So, we recommended that further studies be conducted to detect and evaluate different materials in the implant abutment crown complex. Additional studies are necessary to substantiate our findings in clinical practice. We recommended using lithium disilicate implant abutment in implant-supported prostheses due to its better marginal fit than other tested materials in future studies and resin-ceramic implant abutment due to its better force damping response than other tested materials. Future research could investigate how different surface treatments might impact the marginal fit of materials such as PEEK and resin-ceramic material.

## Conclusions

Lithium disilicate exhibited the smallest marginal gap before and after Cementation, while PEEK showed the largest, followed by Titanium and Resin-ceramic material. All materials experienced an increase in marginal gap values post-cementation, with PEEK and Titanium showing the most significant changes. Resin-ceramic material had the highest shock absorption for force damping, followed by PEEK and Lithium disilicate, while Titanium recorded the highest impact force, indicating the least damping ability. Lithium disilicate ensures superior marginal adaptation, while Resin-ceramic material enhances stress absorption, highlighting the importance of material selection in optimizing implant-supported restorations.

## Data Availability

The datasets used and/or analyzed during the current study are available from the corresponding author on reasonable request.

## Abbreviations

STL: Standard Tessellation Language file
CAD/CAM: Computer-aided design/computer-aided manufacturing
PEEK: Poly-ether-ether-ketone
CEREC: Chair-side Economical Restoration of Esthetic Ceramic
